# CSL112 (Apolipoprotein A-I [Human]) Enhances Cholesterol Efflux Similarly in Healthy Individuals and Stable Atherosclerotic Disease Patients

**DOI:** 10.1161/ATVBAHA.118.310538

**Published:** 2018-02-08

**Authors:** Andreas Gille, Denise D’Andrea, Michael A. Tortorici, Gunter Hartel, Samuel D. Wright

**Affiliations:** From the CSL Limited, Parkville, Australia (A.G.); CSL Behring, King of Prussia, PA (D.D., M.A.T., S.D.W.); and QIMR Berghofer Medical Research Institute, Brisbane City, Australia (G.H.).

**Keywords:** apolipoproteins, atherosclerosis, clinical trial, coronary disease, high density lipoprotein-1

## Abstract

**Objective—:**

CSL112 (apolipoprotein A-I [apoA-I; human]) is a novel formulation of apoA-I in development for reduction of early recurrent cardiovascular events after acute myocardial infarction. Cholesterol efflux capacity (CEC) is a marker of high-density lipoprotein (HDL) function that is strongly correlated with incident cardiovascular disease. Impaired CEC has been observed in patients with coronary heart disease. Here, we determined whether infused apoA-I improves CEC when administered to patients with stable atherosclerotic disease versus healthy volunteers.

**Approach and Results—:**

Measurements of apoA-I, HDL unesterified cholesterol, HDL esterified cholesterol, pre–β1-HDL, and CEC were determined in samples from patients with stable atherosclerotic disease before and after intravenous administration of CSL112. These measures were compared with 2 prior studies in healthy volunteers for differences in CEC at baseline and after CSL112 infusion. Patients with stable atherosclerotic disease exhibited significantly lower ATP-binding cassette transporter 1–mediated CEC at baseline (*P*<0.0001) despite slightly higher apoA-I levels when compared with healthy individuals (2 phase 1 studies pooled; *P*≤0.05), suggesting impaired HDL function. However, no differences were observed in apoA-I pharmacokinetics or in pre–β1-HDL (*P*=0.5) or CEC (*P*=0.1) after infusion of CSL112. Similar elevation in CEC was observed in patients with low or high baseline HDL function (based on tertiles of apoA-I–normalized CEC; *P*=0.1242). These observations were extended and confirmed using cholesterol esterification as an additional measure.

**Conclusions—:**

CSL112 shows comparable, strong, and immediate effects on CEC despite underlying cardiovascular disease. CSL112 is, therefore, a promising novel therapy for lowering the burden of atherosclerosis and reducing the risk of recurrent cardiovascular events.

## Abstract 2

Supplemental Digital Content is available in the text.

Despite advances in treatment of acute myocardial infarction (AMI), patients experience a high risk of reinfarction and cardiovascular death in the weeks immediately after the index event. For example, data from the Plato trial show that approximately half of the new major adverse cardiovascular events in the year after AMI occur in the first month.^1^ At least half of these events occur in nonculprit arteries,^2^ presumably through the rupture or erosion of the diffuse plaque that characterizes coronary atherosclerosis. Thus, a systemic reduction of plaque burden may offer an attractive means of reducing risk.

Cholesterol is known to be removed from the lipid-rich atherosclerotic plaque and transported to the liver by the action of apolipoprotein A-I (apoA-I), the primary functional component of high-density lipoprotein (HDL). The first step in this process is the efflux of cholesterol from plaque macrophages to lipid-poor apoA-I. Consistent with this notion, the risk of symptomatic coronary heart disease (CHD) in large patient cohorts is strongly predicted by ex vivo measures of cholesterol efflux capacity (CEC) of plasma,^3–6^ see Brownell and Rohatgi^7^ 2016 for review. It has been hypothesized that agents that act to increase CEC should promote removal of cholesterol from plaque, reduce plaque burden, and potentially reduce the risk of CHD events. This has been referred to as the cholesterol efflux hypothesis.^8^

CSL112 (apoA-I [human]) is plasma-derived apoA-I reconstituted into disk-shaped lipoproteins with phosphatidylcholine.^9^ Prior work has shown that infusion of CSL112 causes a robust elevation of CEC,^10–12^ and an ongoing development program will test the ability of this agent to reduce the risk of early recurrent cardiovascular events in patients with AMI, thereby testing the cholesterol efflux hypothesis.

Data from healthy individuals indicate that infusion of CSL112 causes a strong and rapid elevation in CEC.^10^ Nevertheless, patients with cardiovascular disease present a unique challenge. Published work suggests that the clearance of endogenous apoA-I may be accelerated in patients with risk factors for CHD.^13^ More importantly, several authors have reported that the HDL from patients with CHD may have impaired functionality and have further speculated that the physiological status of these patients may alter the HDL to render it less functional or even dysfunctional.^14^ For example, HDL purified from patients with stable atherosclerotic disease exhibits lower CEC than that from healthy subjects even after normalization for protein or cholesterol content.^15,16^ Recent work using both animal and human subjects suggests that the function of HDL may be impaired by the action of serum amyloid A, an HDL-associated serum protein that is upregulated in acute coronary syndrome, CHD, and any condition that provokes a hepatic acute phase response.^17,18^ It is thus possible that the amount or functional activity of CSL112 might be altered in patients with cardiovascular disease.

Here, we report the pharmacokinetics and pharmacodynamics of CSL112 in patients with stable atherosclerotic disease and compare these data with findings from prior work with healthy volunteers without evidence of CHD.

## Materials and Methods

### Study Design

The study designs of the phase 1 single (NCT01129661) and phase 1 multiple (NCT01281774) ascending dose (SAD and MAD) studies have been described in Easton et al^19^ and Gille et al^10^ and the phase 2a (NCT01499420) in Tricoci et al.^12^

The SAD study was a randomized, double-blind, placebo-controlled investigation performed at Royal Adelaide Hospital, Adelaide, Australia. The MAD study was a randomized, placebo-controlled, sponsor-unblinded study performed at Q-Pharm, Brisbane, Australia. The phase 2a study was a randomized, double-blind, placebo-controlled, multicenter study performed at 10 sites in the United States.

The primary objective of all 3 studies was to evaluate the safety and tolerability of escalating doses of CSL112 after single or multiple intravenous infusions, together with an evaluation of the pharmacokinetics of apoA-I after single and multiple intravenous infusions of CSL112, and the pharmacodynamics effects linked to CSL112.

### Inclusion and Exclusion Criteria

The main inclusion criteria in the phase 1 studies were male or female healthy subjects aged 18 to <55 years, weighing at least 45 kg in the SAD study and 50 kg in the MAD study, capable of understanding the purposes and risks of the study, and able to provide written informed consent. Subjects were excluded from the studies if they had significant medical conditions, any clinically relevant abnormal laboratory values, history of coagulopathy, hypotension, evidence of renal impairment, evidence of substance or alcohol abuse, or were unable to comply with the study protocol.

In the phase 2a study, subjects were male or female aged 18 to <80 years weighing at least 50 kg. Subjects had to have a history of atherosclerotic coronary artery disease/surgical revascularization (prior AMI, percutaneous coronary intervention, or coronary artery bypass graft surgery) or prior percutaneous or surgical peripheral revascularization procedure. One month or longer must have elapsed between a subject’s randomization and any acute event, revascularization procedure, or hospitalization for chest pain. Subjects were required to be on dual antiplatelet medication, including 75−325 mg/d aspirin and a P2Y12 inhibitor (75 mg/d clopidogrel or 10 mg/d prasugrel). In addition, subjects were stratified in equal proportions to have normal renal function (creatinine clearance ≥90 mL/min) or mild renal impairment (creatinine clearance ≥60 and <90 mL/min).

Subjects in all 3 studies provided written informed consent before any study-specific assessments were performed. The studies were conducted in accordance with standards of Good Clinical Practice, as defined by the International Conference on Harmonization, the principles outlined in the Declaration of Helsinki, and all applicable national and local regulations.

### Randomization

In all 3 studies, subjects were randomized to receive either CSL112 or placebo in the approximate ratio of 3:1 (CSL112:placebo).

### Study Product, Dose, and Administration

CSL112 is apoA-I purified from human plasma and reconstituted with phosphatidylcholine to form lipoprotein particles suitable for intravenous infusion. It is similar in size, structure, and function to nascent HDL particles.^9^ Lyophilized CSL112 presented in glass vials was dissolved in sterile water for injection and dosed based on the protein content. In all 3 studies, the placebo comprised 0.9% sodium chloride solution for injection. CSL112 or placebo was administered intravenously via a 2-hour infusion. The study product was physically masked to maintain blinding. Unblinded site personnel prepared and administered the study product.

In the SAD study, subjects were stratified by body weight and received either placebo or 1 of 6 different CSL112 doses: 5, 15, 40, 70, 105, and 135 mg/kg. In the MAD study, subjects received placebo or CSL112 either once weekly (1 W) or twice weekly (2 W) for 4 weeks: 3.4 g or 6.8 g 1 W CSL112 or placebo (a total of 4 infusions); 3.4 g 2 W CSL112 or placebo (a total of 8 infusions). In the phase 2a study, subjects received placebo or 1.7, 3.4, or 6.8 g CSL112.

### ApoA-I Pharmacokinetic Assessment

ApoA-I was assessed at a specialty lipid laboratory (Pacific Biomarkers, Seattle, WA) by an immunonephelometric method run on Roche Modular P. Blood samples for pharmacokinetics assessment were collected at time points selected to capture the peak and expected decline in apoA-I plasma concentration. The pharmacokinetics samples in the SAD study were collected on days 1 to 4, 6, 8, and 11. In the MAD study, blood samples for pharmacokinetics analysis were taken on days 1 to 4, 6, 8, 9, 15, 21 to 25, and 27 for 1 W dosing groups and on days 1 to 4, 8, 11, 15, 18, 22, and 25 to 28 for the 2 W dosing group. In the phase 2a study, blood samples for pharmacokinetics analysis were taken on days 1 to 7. Validation of pharmacokinetics bioanalysis has been described in detail.^19^

Pharmacokinetics parameters for baseline-corrected plasma concentrations of apoA-I were determined after single and multiple intravenous infusions of CSL112 and included area under the plasma concentration time curve from time point zero (before dosing) to the last time point above baseline (AUC_0–last_), maximum plasma concentration, and half-life.

To compare the pharmacokinetics across the studies, a pooled population pharmacokinetics analysis was conducted using nonlinear mixed effects modeling software (NONMEM, version 7.2). A 2-compartment intravenous model parametrized in terms of elimination clearance, central volume of distribution (Vc), intercompartmental clearance (Q), peripheral volume of distribution (Vp), and baseline apoA-I serum concentration. Random effects were assumed to be distributed normally with mean 0 and variance ω^2^. Residual error was included as an additive and proportional error model. Covariates were tested in the model using a stepwise forward addition and backward elimination approach. Covariates that were tested included body weight, age, sex, creatinine clearance, dose amount, and study population (healthy subjects versus patients with stable atherosclerotic disease). Covariates were determined to be statistically significant in the model fit as estimated by the likelihood ratio test. The objective function value in NONMEM is equal to −2 log likelihood, thus the likelihood ratio test (the test statistic is a χ^2^ distribution) of 2 nested models with or without a covariate was constructed based on the difference in objective function value between 2 models. The significance level for the forward addition approach was determined with a decrease of 3.84 (*P*<0.05; *df*=1) while the significance level of backward deletion step was assessed on the level of an increase in objective function value of 10.83 (*P*<0.001; *df*=1). Models were evaluated using standard diagnostic plots and evaluation of residuals versus model predictions and over time.

### Pharmacodynamic Biomarkers

The following biomarkers were assessed in the SAD, MAD, and phase 2a studies: apoA-I, ATP-binding cassette transporter 1 (ABCA1)–dependent, ABCA1-independent, and total CEC, pre–β1-HDL, apolipoprotein B (apoB), triglycerides, total cholesterol, and HDL cholesterol (HDL-C). HDL unesterified cholesterol (HDL-UC) and HDL esterified cholesterol (HDL-EC) were measured in the SAD and phase 2a studies only.

Cholesterol efflux assays were performed in apoB-depleted serum samples using J774 macrophages at Vascular Strategies LLC, Plymouth Meeting, PA, as previously described.^20^ All other pharmacodynamics biomarkers were assessed in plasma samples at Pacific Biomarkers. Pre–β1-HDL was preserved by 21-fold dilution in 50% sucrose before freezing and measured by ELISA (Sekisui Medical Co, Tokyo, Japan). HDL was separated by the polyethylene glycol precipitation method. All cholesterol and triglycerides were measured by standard enzymatic methods. Validation of pharmacodynamics assays was conducted ahead of the assessment of clinical samples to ensure accuracy of reported concentrations and activities in the presence of CSL112.^10^

In the studies, no food was provided for 8 hours before and after dosing, and all data shown were obtained in fasting samples. During the SAD study, blood samples for assessing total cholesterol, HDL-UC, HDL-EC, apoB, and triglycerides were collected at 0 hour (before infusion), 1, 2, 4, 8, 24, 48, 72, and 168 hours. Pre–β1-HDL and CEC were assessed in blood samples taken (at 0 hour [before infusion], 2, 8, 24, 48, 72, and 168 hours]. In the MAD study, apoB samples were taken at the same time points irrespective of 1 W or 2 W dosing, that is, before dosing on days 1, 8, 15, 22, and on day 28. Samples for measurement of CEC and pre–β1-HDL were taken on day 1 (before dosing, at 2, 4, and 8 hours), day 2, day 22 (before dosing and 2, 4, and 8 hours), and day 23 in the 1 W dosing groups. For the 2 W dosing group, these samples were collected on day 1 (before dosing and at 2, 4, and 8 hours), day 2, day 25 (before dosing and 2, 4, and 8 hours), and day 26. In the phase 2a study, blood samples for assessing total cholesterol, HDL-UC, HDL-EC, apoB, and triglycerides were collected at 0 hour (before infusion), 2, 4, 8, 24, 48, 72, 96, and 144 hours. Pre–β1-HDL and CEC were assessed in blood samples taken at 0 hour (before infusion), 2, 4, 8, 24, 48, and 72 hours.

### Pharmacodynamic Parameters

Pharmacodynamic parameters were calculated from biomarker concentrations and activities. The following pharmacodynamic parameters were assessed: area under the effect curve for time point zero to the time point of the last quantifiable concentration/activity (AUEC_0–last_), maximum biomarker concentration/activity (R_max_), and time to reach maximum concentration/activity (T_max_).

### Statistical Analysis

The sample size was not based on formal power calculations because no formal hypotheses were to be tested. The minimal sample size per dose group in each study was selected as the smallest possible number to allow a decision on dose escalation to be made. In the SAD study, 42 subjects received CSL112 and 15 subjects received placebo. In the MAD study, 27 subjects received CSL112 and 9 subjects received placebo. In the phase 2a study, 33 subjects received CSL112 (7 received 1.7 g, 12 received 3.4 g, and 14 received 6.8 g) and 11 subjects received placebo. Subjects were randomized by an individual not directly involved in the analysis of study results. A randomization block size of 4 (3 CSL112:1 placebo) was used to ensure the balance between dosing groups was maintained.

The data analysis of the study data involved descriptive statistics because there was no formal hypothesis to be tested during the study. The placebos from each cohort were combined to form a single placebo group for each study.

The analysis of apoA-I–normalized CEC was performed as a post hoc analysis across the 3 clinical trials. For each subject and each time point that assessments of apoA-I and CEC were conducted, the ratios of total CEC:apoA-I and ABCA1-dependent CEC:apoA-I were calculated as the normalized CEC activity of apoA-I. Summary statistics calculated were means and SDs. Comparisons of baseline biomarker activities between clinical studies used unpaired *t* test. Comparison of post-treatment versus baseline results was analyzed using paired *t* tests or other repeated measures analyses. Comparison of the relationship of total CEC AUEC_0–24_, pre–β1-HDL AUEC_0–24_, and HDL-C AUEC_0─72_ versus apoA-I AUC between studies was done using random effects regression models with patient as the random effect and testing the parallelism of slopes hypothesis. A similar analysis was used to compare the slopes of CEC regressed onto CSL112 dose split by tertiles of baseline apoA-I–normalized CEC (Figure 6).

The baseline value for all analyses was the last value recorded before dosing on day 1 (ie, before administration of study medication). Pharmacokinetics and pharmacodynamics baseline correction involved subtracting the baseline value from the value obtained from each sample collected after study medication administration. The length of elevation and comparison of post-dose versus baseline concentration in pre–β1-HDL were assessed by paired *t* test.

SAS version 9.2 (SAS Institute Inc, Cary, NC) was used for the analysis of study data. Noncompartmental pharmacokinetics analysis was performed using model 202 (constant infusion) in WinNonlin Enterprise version 5.2 (Pharsight Corporation, Montreal, Canada). Noncompartmental pharmacodynamics analysis of biomarkers was performed using Model 220 (baseline effect) in WinNonlin Enterprise version 5.2. Post hoc analyses were performed using JMP-Pro 13.2 (SAS Institute Inc, Cary, NC).

## Results

### Demographics and Baseline Characteristics

As previously described, 44 patients with stable atherosclerotic disease who were receiving standard-of-care therapy, including aspirin and either clopidogrel or prasugrel, were studied in a phase 2a trial (NCT01499420).^12^ Patients with stable atherosclerotic disease were defined as subjects with a history of atherosclerotic coronary artery disease/surgical revascularization or prior percutaneous or surgical peripheral revascularization procedure, who were randomized at least 1 month or more after any acute event, revascularization procedure, or hospitalization for chest pain.

Baseline characteristics of these patients are compared in Table 1 with those of healthy subjects studied in 2 prior phase 1 trials with CSL112 (NCT01129661 and NCT01281774).^10,19^ Fifty-seven and 36 healthy subjects were included in the phase 1 SAD and MAD studies, respectively. Baseline parameters in the phase 1 SAD and MAD studies were similar for most parameters (*P*>0.05) with the exception of body mass index (*P*=0.0397) and ABCA1-independent CEC (*P*=0.0025). As expected, patients with atherosclerotic disease were older, had a greater body mass index, and had higher levels of apoB and triglycerides than the healthy subjects. High sensitivity C-reactive protein levels were measured only in the phase 2a population (Table 1) and showed levels corresponding to a medium cardiovascular risk.^21^

**Table 1.**
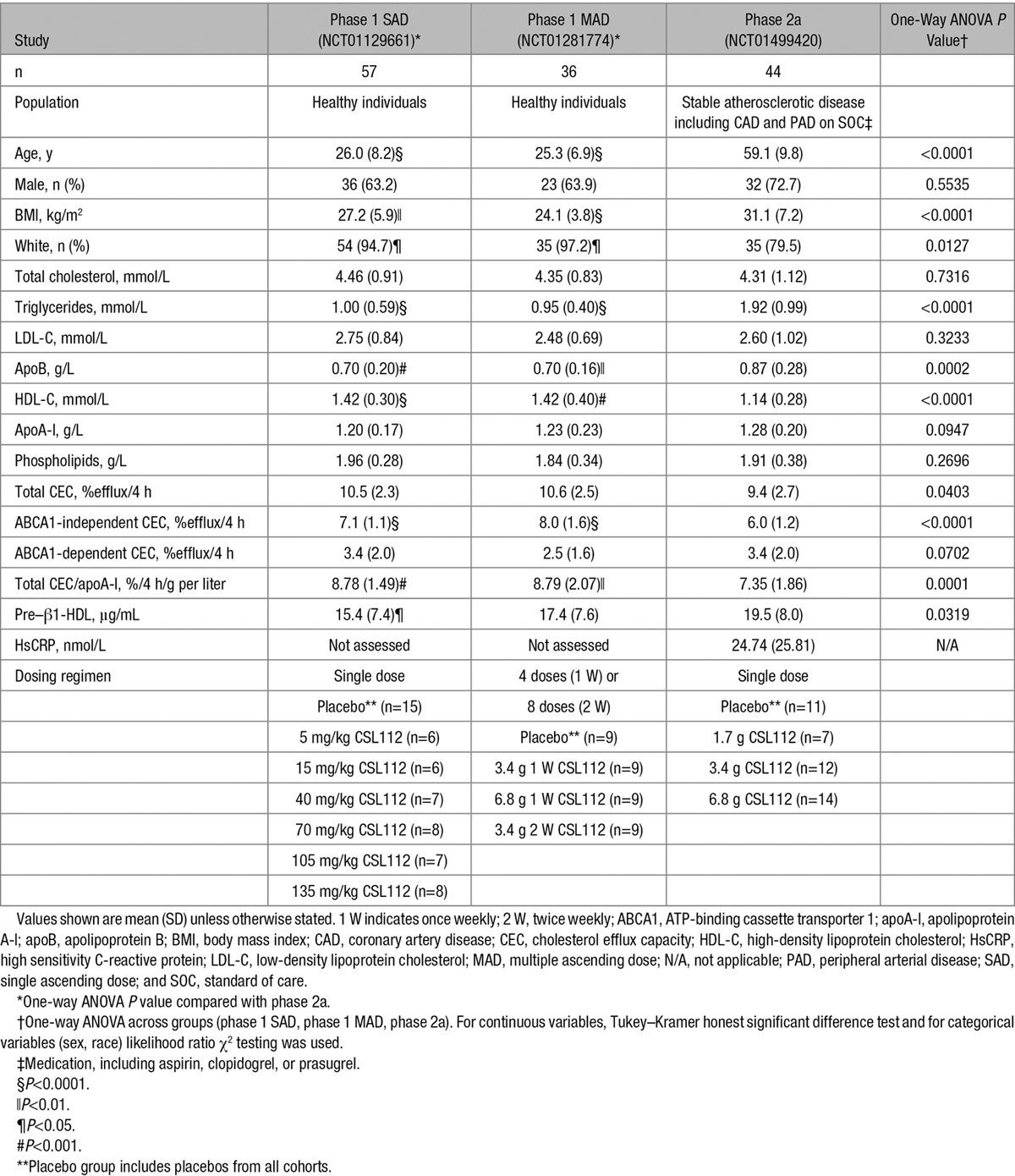
Summary of Demographics and Baseline Characteristics of Patients With Atherosclerotic Disease Compared With Healthy Volunteers in 2 Prior Studies With CSL112

### Pharmacokinetics of ApoA-I

In the phase 1 SAD study, CSL112 dosing was weight based. Because of an influence of body weight on the clearance of apoA-I, pharmacokinetics modeling of the phase 1 SAD study data predicted that fixed doses would result in less variability in apoA-I exposure at extremes of the body weight range.^19^ Consequently, fixed doses of CSL112 were used in the phase 1 MAD and phase 2a studies.

CSL112 is apoA-I from human plasma and cannot be distinguished from endogenous apoA-I.

**Figure 1.**
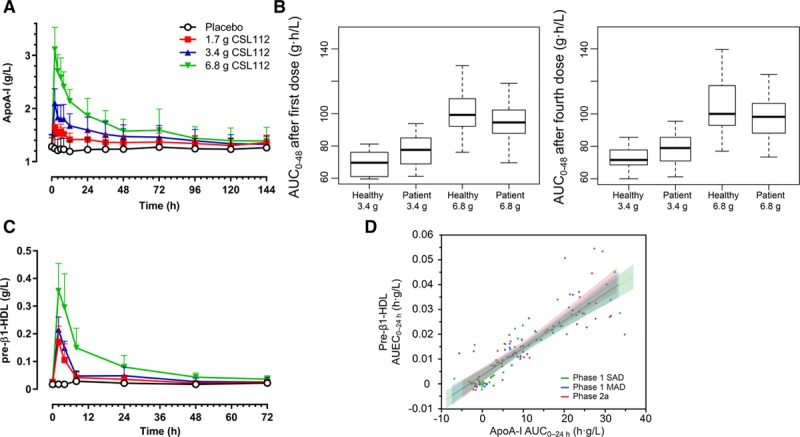
Apolipoprotein A-I (apoA-I) pharmacokinetic profile after infusion of CSL112. Infusions started at time=0 h and lasted for 2 h. **A**, Shown are mean+SD unadjusted total plasma apoA-I concentrations. **B**, Predicted exposure (area under the curve 0−48 h [AUC_0−48_]) in healthy subjects and patients with stable atherosclerotic disease based on population pharmacokinetic modeling. **C**, Shown are mean+SD unadjusted pre–β1-high-density lipoprotein (HDL) concentrations, measured by ELISA (Sekisui, Japan), in the phase 2a study. **D**, The relationship of baseline-corrected apoA-I exposure (AUC_0−24_) and baseline-corrected pre–β1-HDL exposure (area under the effect curve 0−24 h [AUEC_0−24_]) is shown in healthy volunteers (phase 1 single ascending dose [SAD] and multiple ascending dose [MAD] studies) and in a phase 2a study in stable atherosclerotic disease for comparison. Shown are linear regression lines and 95% CIs for each study, calculated using a random effects regression model with patient as the random effect and testing the parallelism of slopes hypothesis (difference in slopes, *P*=0.5).

CSL112 pharmacokinetics parameters were, therefore, determined from baseline-corrected plasma concentrations of apoA-I. As previously reported in healthy subjects^19^ and patients with atherosclerotic disease,^12^ the pharmacokinetics of apoA-I is characterized by dose-dependent increases in concentration (T_max_ ≈2 hours) and a slow elimination (Figure 1A). A pooled population pharmacokinetics analysis revealed that a 2-compartment model described the pharmacokinetics of apoA-I concentration versus time profiles well. After accounting for the effect of body weight on apoA-I clearance in the model, there was no significant effect of population (healthy subject versus patients with stable atherosclerotic disease) or other demographics that were tested in the model on the pharmacokinetics of apoA-I (manuscript in preparation).^22^ This is further demonstrated in Figure 1B, which shows the similar model predicted exposure (AUC, 0–48 hours) in healthy subjects and patients. The pharmacokinetics of CSL112 in healthy subjects (in the MAD study) and patients with stable atherosclerotic disease is detailed in Table 2.

**Table 2.**
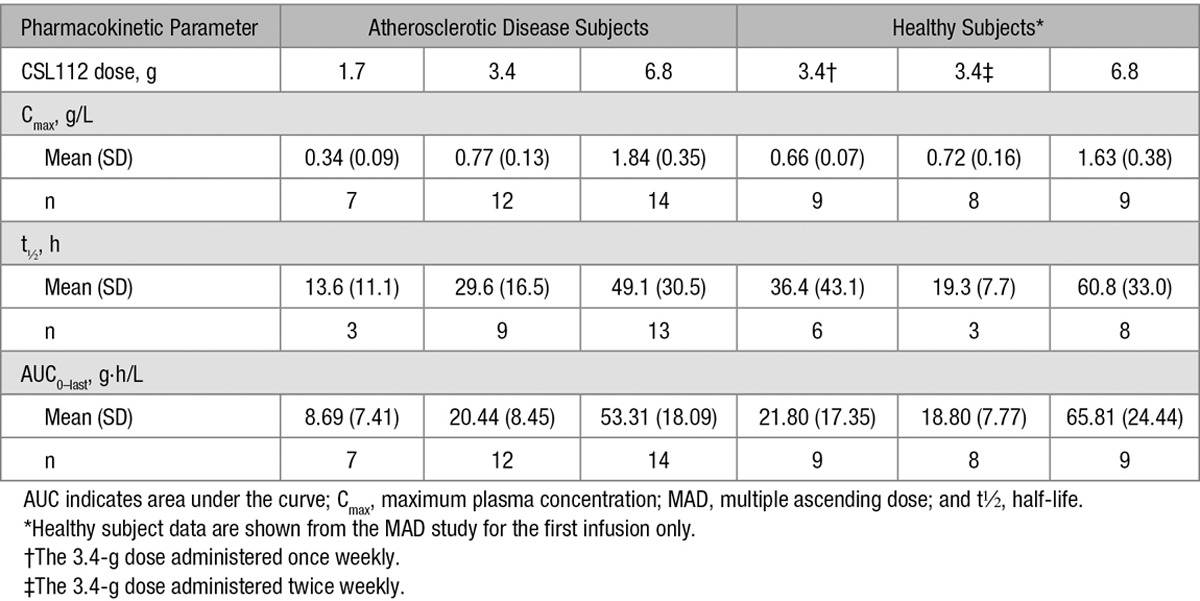
Pharmacokinetic Parameters of Apolipoprotein A-I After Single Infusion of CSL112 in Patients With Stable Atherosclerotic Disease and Healthy Subjects

### CSL112 Causes a Strong Increase in Lipid-Poor ApoA-I (Pre–β1-HDL)

CSL112 when added to blood or plasma undergoes remodeling steps that involve transient fusion with endogenous HDL and rapid fission to yield enlarged HDL and large amounts of lipid-poor apoA-I HDL (pre–β1-HDL).^23^ We measured these small species by ELISA using an anti-pre-β1 antibody.

After the infusion of CSL112, there was an immediate strong elevation in pre–β1-HDL levels. The 6.8 g dose group had the largest fold increase with a 17-fold increase from 0.02 g/L pre-dose to 0.35 g/L after 2 hours. Peak pre–β1-HDL levels were reached at 2-hour post-infusion for all doses (Figure 1C). The duration of elevation was dependent on CSL112 dose: pre–β1-HDL was significantly elevated from baseline at all time points up to 24 hours in the 1.7 g CSL112 dose group (mean±SD: 0.025±0.012 versus 0.035±0.015 g/L [*P*=0.02]), up to 48 hours in the 3.4 g CSL112 dose group (mean±SD: 0.017±0.007 versus 0.027±0.013 g/L [*P*=0.003]), and up to 72 hours in the 6.8 g CSL112 dose group (mean±SD: 0.021±0.006 versus 0.036±0.013 g/L [*P*=0.0001]).

Throughout the dose range in the current study and throughout the hours after infusion, a similar proportion of the infused apoA-I appeared as pre–β1-HDL such that the relationship between apoA-I AUC_0–24_ and pre–β1-HDL area under the effect curve 0 to 24 hours (AUEC_0–24_) seems linear (Figure 1D). Plotting pre–β1-HDL AUEC versus apoA-I AUC allowed us to compare the data from the current study with 2 prior studies of healthy subjects, including an early phase 1 SAD study that used mg/kg dosing. These comparisons showed that the magnitude of elevation of pre–β1-HDL showed no significant differences between healthy subjects and patient populations with stable atherosclerotic disease (difference in slopes; *P*=0.5; Figure 1D). These results suggest that the presence of stable atherosclerotic disease does not affect the clearance of infused CSL112, its interaction with endogenous HDL, or the formation of pre–β1-HDL.

### Esterification of Cholesterol After Movement of Cholesterol From Tissues to HDL

**Figure 2.**
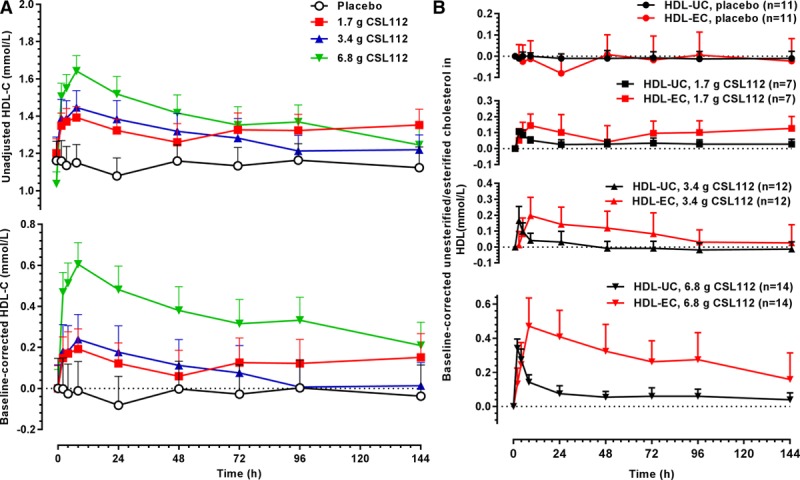
CSL112 infusion causes movement of unesterified cholesterol from tissues to high-density lipoprotein (HDL) and subsequent esterification. **A**, Shown are mean+SE of the mean unadjusted (**top**) and baseline-corrected (**bottom**) HDL cholesterol (HDL-C) concentrations as measured in supernatants in apolipoprotein B–depleted plasma after infusion of CSL112. **B**, Shown are mean+SD for baseline-corrected HDL unesterified cholesterol (HDL-UC) and HDL esterified cholesterol (HDL-EC) per dose group. Peaks in HDL-UC occurred before peaks in HDL-EC.

CSL112 caused a rapid and sustained elevation in HDL-C levels (Figure 2A). This suggests that tissue cholesterol moved to the HDL fraction on infusion of CSL112. The time course for HDL-C showed a peak at 8 hours, and levels remained elevated for at least 72 hours with gradual return to baseline (Figure 2A). A similar pattern was seen also in both phase 1 studies.^10^ To enable comparison between patients with stable atherosclerotic disease and healthy subjects from 2 prior phase 1 studies, HDL-C AUEC_0–__72_ versus apoA-I AUC_0–__72_ was plotted. The elevation in HDL-C after infusion of CSL112 was not significantly different between healthy subjects and patients with stable atherosclerotic disease (difference in slopes; *P*=0.1883; Figure I in the online-only Data Supplement).

The increase in HDL-C observed was modest when compared with the marked increases in apoA-I and pre–β1-HDL. To explore this further, we examined the ratio of HDL-C to apoA-I. We observed that after infusion of CSL112, the HDL-C:apoA-I ratio initially decreased and returned to baseline between 24 and 48 hours post-dose (Figure II in the online-only Data Supplement). This finding is consistent with the large increase in pre–β1-HDL and with the findings of studies of HDL particle size after infusion of CSL112.^23^

**Figure 3.**
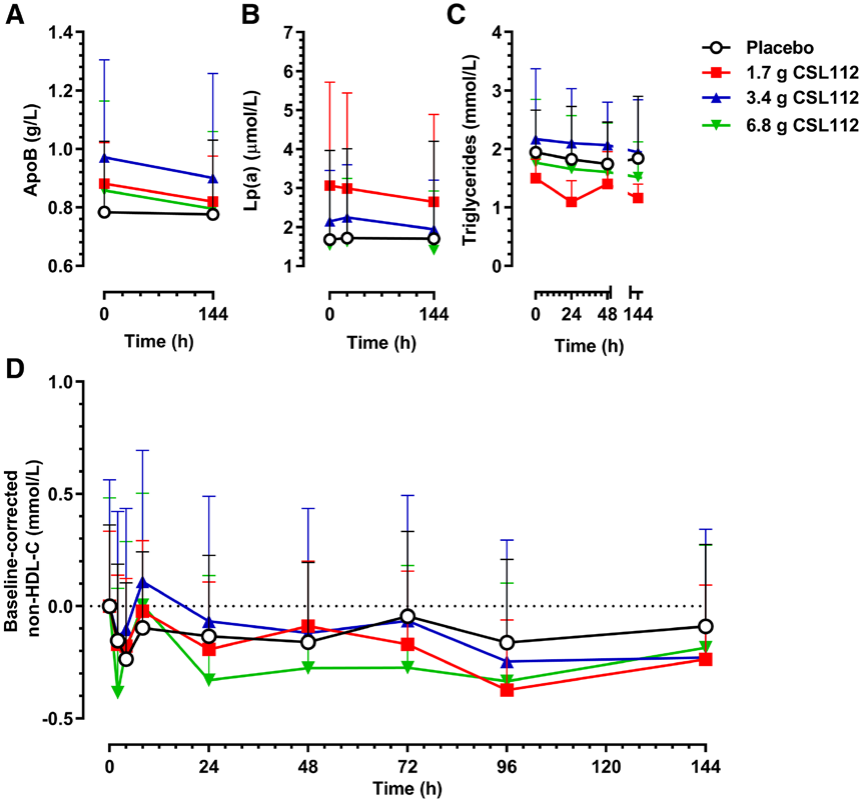
CSL112 causes no increases in apolipoprotein B (apoB), lipoprotein(a) (Lp(a)), triglycerides, or non–high-density lipoprotein cholesterol (HDL-C). Shown are mean+SD of unadjusted data for (**A**) apoB, (**B**) Lp(a), (**C**) triglycerides, and (**D**) baseline-corrected non–HDL-C.

Further assays separately measured the rise in HDL-UC and HDL-EC. The initial rise on infusion was in the HDL-UC fraction, reflecting the well-known ability of UC to exit cells by diffusion and facilitated transport, as well as the well-known inability of EC to exit cells except in chylomicrons. Over the succeeding 8 hours, HDL-UC levels diminished with a quantitatively matching rise in HDL-EC (Figure 2B). Similar results were previously reported in healthy subjects.^10^ CSL112 infusion did not change apoB, lipoprotein(a), triglycerides, or non–HDL-C levels (Figure 3A–3D; Figure III in the online-only Data Supplement).

### Cholesterol Efflux Capacity Ex Vivo

**Figure 4.**
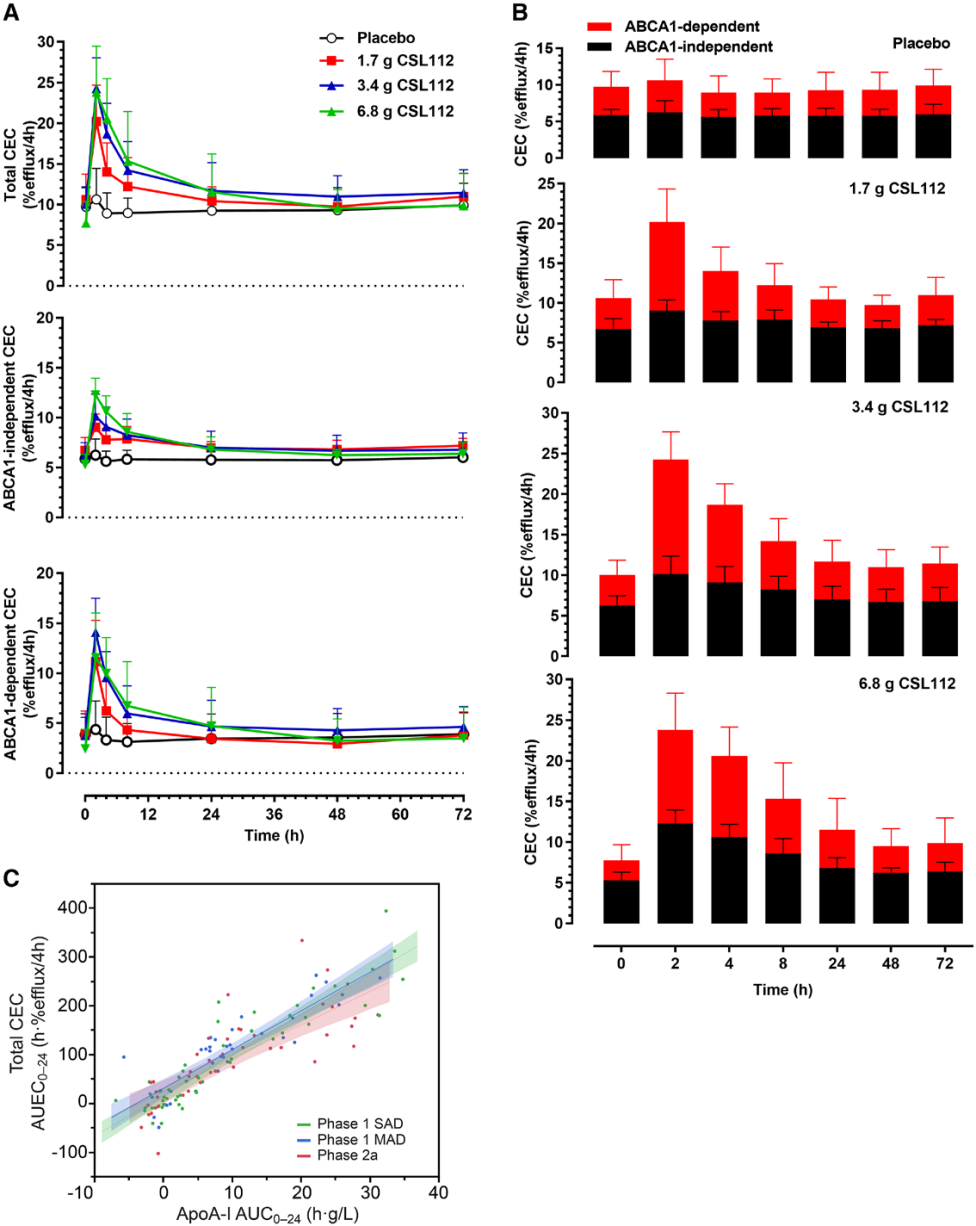
Cholesterol efflux capacity (CEC) after infusion of CSL112. **A**, Shown are time courses (mean+SD) of total CEC (measured in J774 macrophages in presence of cAMP), ATP-binding cassette transporter 1 (ABCA1)–independent CEC (measured in J774 macrophages in absence of cAMP), and ABCA1-dependent CEC (calculated as the difference of ABCA1-independent and total CEC). **B**, Shown are the contributions of ABCA1-dependent and ABCA1-independent efflux to total CEC, by dose group (means+SD). **C**, The relationship of baseline-corrected apolipoprotein A-I (apoA-I) exposure (area under the curve 0−24 h [AUC_0−24_]) and baseline-corrected total cholesterol efflux exposure (area under the effect curve 0−24 h [AUEC_0−24_]) is shown in healthy volunteers (phase 1 single ascending dose [SAD] and multiple ascending dose [MAD] studies) and in a phase 2a study in stable atherosclerotic disease for comparison. Shown are linear regression lines and 95% CIs for each study, calculated using a random effects regression model with patient as the random effect and testing the parallelism of slopes hypothesis (difference in slopes, *P*=0.1).

CEC was measured ex vivo using the method described by de la Llera-Moya et al.^20^ Infusion of CSL112 caused an immediate rise in both the ABCA1-dependent and ABCA1-independent portions of CEC with elevations reaching 2- to 3-fold the baseline levels at high doses of CSL112 (Figure 4A and 4B). Thereafter, CEC followed a gradual decline to baseline between 48 and 72 hours. Analysis of the total CEC AUEC_0–24_ versus apoA-I AUC_0–24_ (Figure 4C) as well as ABCA1-dependent and ABCA1-independent CEC (data not shown) showed these elevations to be linear dose dependent in all 3 study populations. Coincident lines indicate equal elevation in efflux between healthy subjects and patients with CHD (*P*=0.1). This finding suggests that the disease processes that act to reduce the efflux activity of endogenous apoA-I have a negligible effect on infused CSL112.

**Figure 5.**
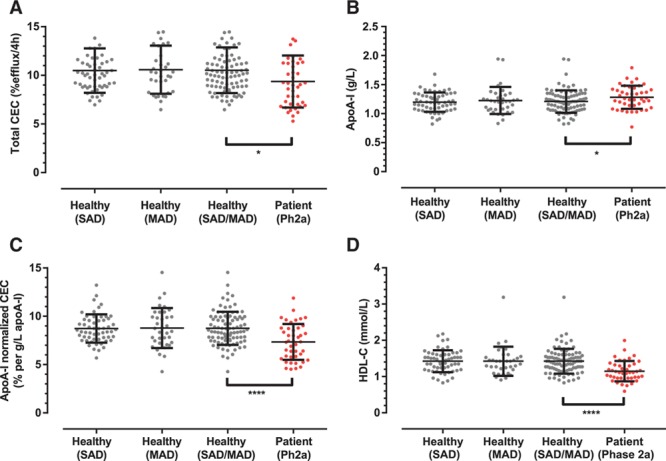
Apolipoprotein A-I (apoA-I)–normalized cholesterol efflux capacity (CEC) discriminates patients with stable atherosclerotic disease and healthy subjects. Shown are individual data points, mean, and SD of baseline (**A**) total CEC, (**B**) apoA-I, (**C**) apoA-I–normalized CEC (calculated as the ratio of total CEC to apoA-I), and (**D**) high-density lipoprotein cholesterol (HDL-C) in healthy subjects participating in the phase 1 single ascending dose (SAD) study, phase 1 multiple ascending dose (MAD) study, the combined group of phase 1 SAD and MAD studies and the patients with stable atherosclerotic disease participating in the phase 2a study. Unpaired *t* test comparing the combined phase 1 SAD and MAD subjects vs phase 2a patients with stable atherosclerotic disease; ****P<0.001; **P<0.01; *P<0.05.

Efflux measures in patients with stable atherosclerotic disease were compared with similar measures in healthy subjects (Figure 5A–5D; Table I in the online-only Data Supplement). As expected from prior studies, baseline total CEC in patients with stable atherosclerotic disease was lower compared with healthy subjects (Figure 5A; *P*<0.05) despite the fact that levels of apoA-I were slightly higher in the patients with stable atherosclerotic disease than in the healthy subjects at baseline (Figure 5B; *P*<0.05). To derive a measure for efflux that is corrected for the apoA-I level (ie, a measure of the specific activity of apoA-I), baseline total CEC was divided by baseline apoA-I. As shown in Figure 5C, patients with stable atherosclerotic disease had lower apoA-I–normalized CEC than healthy subjects, a finding consistent with the proposed HDL dysfunction in patients with CHD (*P*<0.001). Patients with stable atherosclerotic disease also had lower baseline levels of HDL-C (Figure 5D; *P*<0.001) compared with healthy subjects.

**Figure 6.**
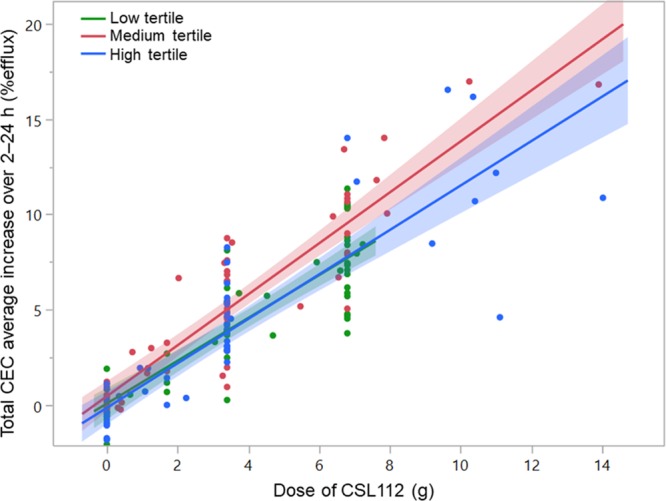
Infusion of CSL112 increases total cholesterol efflux capacity (CEC) regardless of baseline functional apolipoprotein A-I (apoA-I) activity. Participants of phase 1 single ascending dose and multiple ascending dose and phase 2a studies were analyzed as 1 group. Mean total CEC for 2 to 24 h was plotted over dose of CSL112 administered by tertiles of baseline apoA-I–normalized CEC (high-density lipoprotein function)-specific activity at baseline. Shown are individual data points, linear regression lines, and 95% CIs for each tertile calculated using a random effects regression model with tertiles of baseline apoA-I–normalized CEC activity as the random effect and testing the parallelism of slopes hypothesis (difference in slopes; *P*=0.1242).

Having noted that apoA-I–normalized CEC discriminated healthy versus atherosclerotic populations better than either apoA-I or cholesterol efflux alone, we asked if this property at baseline could influence the response of a patient to infusion of CSL112. For the analysis, baseline HDL function (CEC normalized for plasma apoA-I) was calculated for subjects participating in all 3 studies. The data were pooled and stratified into tertiles based on baseline apoA-I–normalized CEC. Total CEC post-infusion (averaged from the end of infusion at 2 to 24 hours post-dose) increased in a dose-dependent manner after infusion of CSL112 and was similarly elevated between subjects in the highest and lowest tertiles of HDL functionality (Figure 6; difference in slopes for tertiles; *P*=0.1242). These data further suggest that elevation of cholesterol efflux by CSL112 is a robust property that is little affected by either baseline HDL functionality or stable atherosclerotic disease.

## Discussion

Previous work with healthy subjects has shown that infusion of CSL112 causes rapid elevation of apoA-I and a subsequent elimination that is similar in half-life to that reported in studies on the half-life of endogenous apoA-I.^19^ Similar slow elimination of infused apoA-I was also seen when CSL112 was infused into patients with stable atherosclerotic disease.^12^ Maximum plasma concentration and AUC_0–last_ show comparable values in healthy subjects and patients with stable atherosclerotic disease, thus demonstrating that underlying disease does not significantly affect the pharmacokinetics of CSL112 as demonstrated in the pooled population pharmacokinetics analysis (manuscript in development). We note that, although the patients with stable atherosclerotic disease in our study showed lower baseline HDL-C than the healthy controls, they did not show lower levels of apoA-I, which have been reported in other studies.^24^

Previous work has also shown that infusion of CSL112 causes movement of cholesterol from tissue into the HDL fraction and that it further promotes the subsequent rapid esterification of effluxed free cholesterol.^9,10^ Because published studies have suggested that patients with stable atherosclerotic disease may have depressed levels of lecithin–cholesterol acyltransferase,^25^ we compared data on these parameters in healthy subjects versus those with stable atherosclerotic disease. In both healthy subjects and patients with stable atherosclerotic disease, the curves tracing HDL-UC fell rapidly from their peak at 2 hours while levels of HDL-EC rose in this time frame, with the curves intersecting between 4 and 6 hours. The apparent precursor–product relationship indicates rapid ongoing conversion of HDL-UC to HDL-EC, known to be mediated by the serum enzyme lecithin–cholesterol acyltransferase. Because of the dynamic nature of cholesterol pools, with continuous clearance from and entry into the HDL pool for both UC and EC, a quantitative measure of lecithin–cholesterol acyltransferase cannot be derived from these data. Nevertheless, it is apparent that esterification of cholesterol in response to infusion of CSL112 is similar in healthy subjects and patients with stable atherosclerotic disease because the intersection point of the falling HDL-UC curve with the rising HDL-EC curve occurs at similar intervals after infusion (4.6–6.4 hours for healthy subjects,^10^ 4.2–4.4 hours for stable atherosclerotic disease).

HDL-C levels are only loosely associated with the ability of plasma to either promote cholesterol efflux from cells or to reduce inflammatory responses of cells ex vivo.^14^ A potential explanation of this phenomenon is the observation that HDL may lose functional activity in some disease states resulting in dysfunctional or poorly functional HDL. We have used a novel measure of functionality: apoA-I–normalized CEC (the ratio of cholesterol efflux to apoA-I), to assess this. Consistent with prior observations of impaired functionality in patients with CHD,^15,16^ we find that patients with stable atherosclerotic disease have lower apoA-I–normalized CEC than healthy subjects.

The molecular features that cause the reduction in HDL function in cardiovascular disease are unknown, and we could not predict on first principles whether newly infused apoA-I would be fully functional or if it would take on the compromised functional activity of the host. Here, we show that CSL112 elevates cholesterol efflux in a profound and dose-dependent fashion and that the magnitude and duration of this elevation are independent of the disease state of the subject. We documented this with 2 types of comparisons. In the first, we compared healthy subjects versus those with diagnosed cardiovascular disease, and in the second, we compared an aggregate of healthy and diseased patients by tertiles of baseline HDL function (apoA-I–normalized CEC). Both comparisons showed equivalent elevation of efflux by CSL112, regardless of disease status or baseline HDL functionality. These data suggest that infusions of CSL112 are likely to be fully functional even in patients with dysfunctional endogenous HDL. Finally, we note that subjects with stable atherosclerotic disease had a significantly higher level of triglycerides compared with healthy subjects at baseline (*P*<0.0001) yet showed equivalent elevation of CEC. Thus, we have demonstrated that the presence of elevated plasma triglycerides did not interfere with pharmacodynamics effects of CSL112.

There are several limitations to this analysis. First, data on patients with stable atherosclerotic disease and healthy subjects were collected in different studies, and so comparisons drawn are from post hoc analyses. Second, it could be anticipated that the results of this study may not be applicable to post-AMI patients who may exhibit strongly increased markers of inflammation that could impair HDL function.^16^ Any such effect of inflammation on the response to CSL112 is likely to be low given that infusion of CSL112 in a phase 2b trial of post-AMI patients caused a dramatic increase in CEC.^11^ However, a formal analysis and comparison with healthy subjects have not yet been conducted.

Cholesterol efflux is a important parameter favoring development of CSL112 for reduction of early recurrent events after an AMI. Depressed levels of cholesterol efflux are associated with higher rates of CHD^3–5^ and an increased risk of cardiovascular events in subjects with CHD.^26,27^ A recent study has also correlated the improved cardiovascular outcome associated with Mediterranean diet^28^ with improvements in cholesterol efflux.^29^ Importantly, efflux values to apoB-depleted plasma are much stronger predictors of risk of CHD than HDL-C values.^3–5^ The recent AEGIS-I study (ApoA-1 Event Reducing in Ischemic Syndromes) has now shown that CSL112 has a satisfactory safety profile when initiated in the days after an AMI and that it produces a marked increase in CEC in the setting of AMI.^11^ The ability of CSL112 to strongly elevate efflux, even in patients with atherosclerotic disease, suggests that it may be a promising agent for rapidly removing plaque cholesterol and reducing the risk of early recurrent atherothrombotic events.

## Acknowledgments

We thank Kate Holliday of Meridian HealthComms, Plumley, UK, for providing editorial support, which was funded by CSL Behring, King of Prussia, PA, in accordance with Good Publication practice (GPP3).

## Sources of Funding

The clinical studies included in this study were funded by CSL Limited.

## Disclosures

All authors are or were employees of CSL Limited or CSL Behring.

## Supplementary Material

## Notes

Highlights

- Patients with stable atherosclerotic disease had a lower baseline cholesterol efflux capacity compared with healthy subjects, consistent with notion of dysfunctional high-density lipoprotein in patients with coronary heart disease.
- Infusion of CSL112, a novel formulation of human apolipoprotein-I, dramatically elevated lipid-poor apolipoprotein-I (pre–β1-high-density lipoprotein) levels and enhanced cholesterol efflux capacity to a similar extent in patients with stable atherosclerotic disease and healthy subjects.
- Esterification of cholesterol in response to infusion of CSL112 was also similar in patients with stable atherosclerotic disease and healthy subjects.
- The ability of CSL112 to enhance cholesterol efflux capacity was not influenced by baseline high-density lipoprotein functionality or the presence of stable atherosclerotic disease.
