# Supplementary material for: CSL112 (Apolipoprotein A-I [Human]) Enhances Cholesterol Efflux Similarly in Healthy Individuals and Stable Atherosclerotic Disease Patients
Source: Arterioscler Thromb Vasc Biol. 2018 Feb 8;38(4):953–63. doi: 10.1161/ATVBAHA.118.310538 (PMC5895137; doi:10.1161/ATVBAHA.118.310538)

Supplement Material

Supplementary Figure I. HDL-cholesterol profile following infusion of CSL112

The relationship of baseline-corrected apolipoprotein A-I (apoA-I) exposure (area under the curve 0–72 h [$AUC_{0–72}$]) and baseline-corrected HDL-cholesterol (HDL-C) exposure (area under the effect curve 0–72 h [$AUEC_{0–72}$]) is shown in healthy volunteers (Phase 1 SAD and MAD studies) and in a Phase 2a study in stable atherosclerotic disease for comparison. Shown are linear regression lines and 95% confidence interval for each study, calculated using random effects regression models with patient as the random effect and testing the parallelism of slopes hypothesis (difference in slopes, p=0.1883).

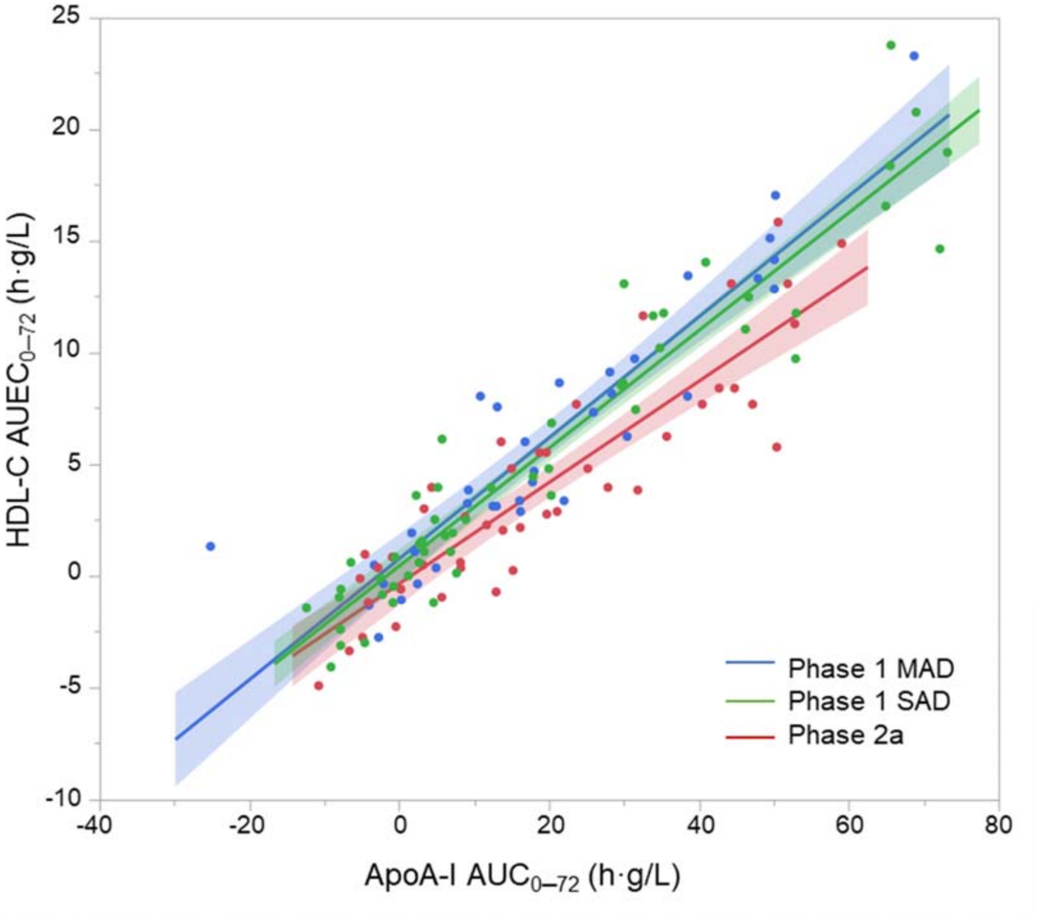

Supplementary Figure II. HDL-cholesterol:apolipoprotein A-I ratio over time

Shown are mean ± standard deviation of the ratio of HDL-cholesterol (HDL-C) to apolipoprotein A-I (apoA-I) in stable atherosclerotic disease patients participating in the Phase 2a study.

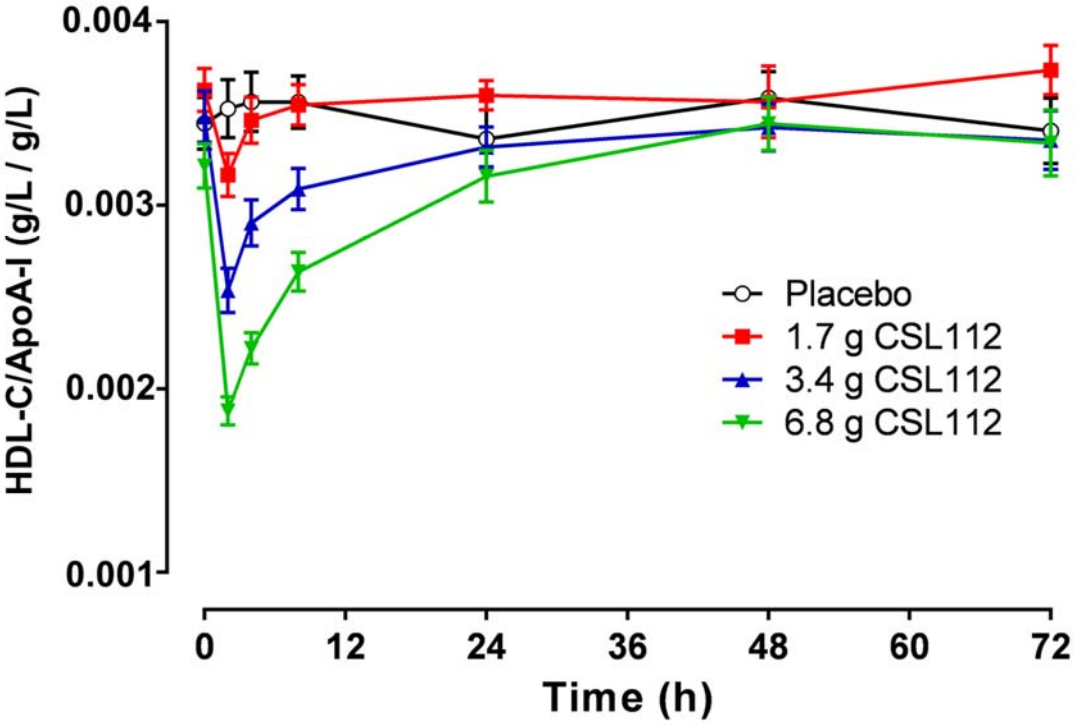

Supplementary Figure III. CSL112 does not increase non-HDL-cholesterol.

Shown are mean + standard deviation of unadjusted non-HDL-cholesterol.

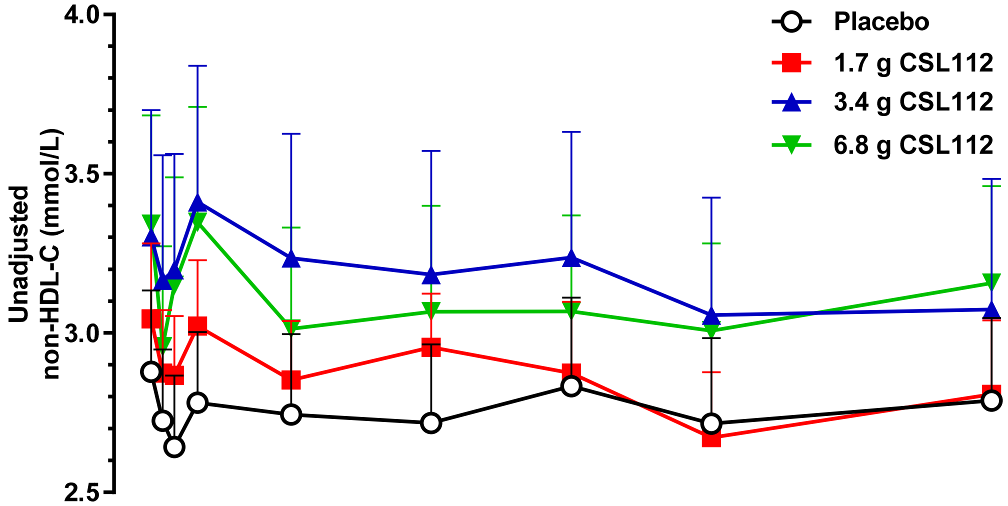

Supplement: Supplementary file 1 [file atv-38-953-s001.pdf]
