# Supplementary material for: CSL112 (Apolipoprotein A-I [Human]) Enhances Cholesterol Efflux Similarly in Healthy Individuals and Stable Atherosclerotic Disease Patients
Source: Arterioscler Thromb Vasc Biol. 2018 Feb 8;38(4):953–63. doi: 10.1161/ATVBAHA.118.310538 (PMC5895137; doi:10.1161/ATVBAHA.118.310538)
Supplement: Supplementary file 2 [file atv-38-953-s002.pdf]

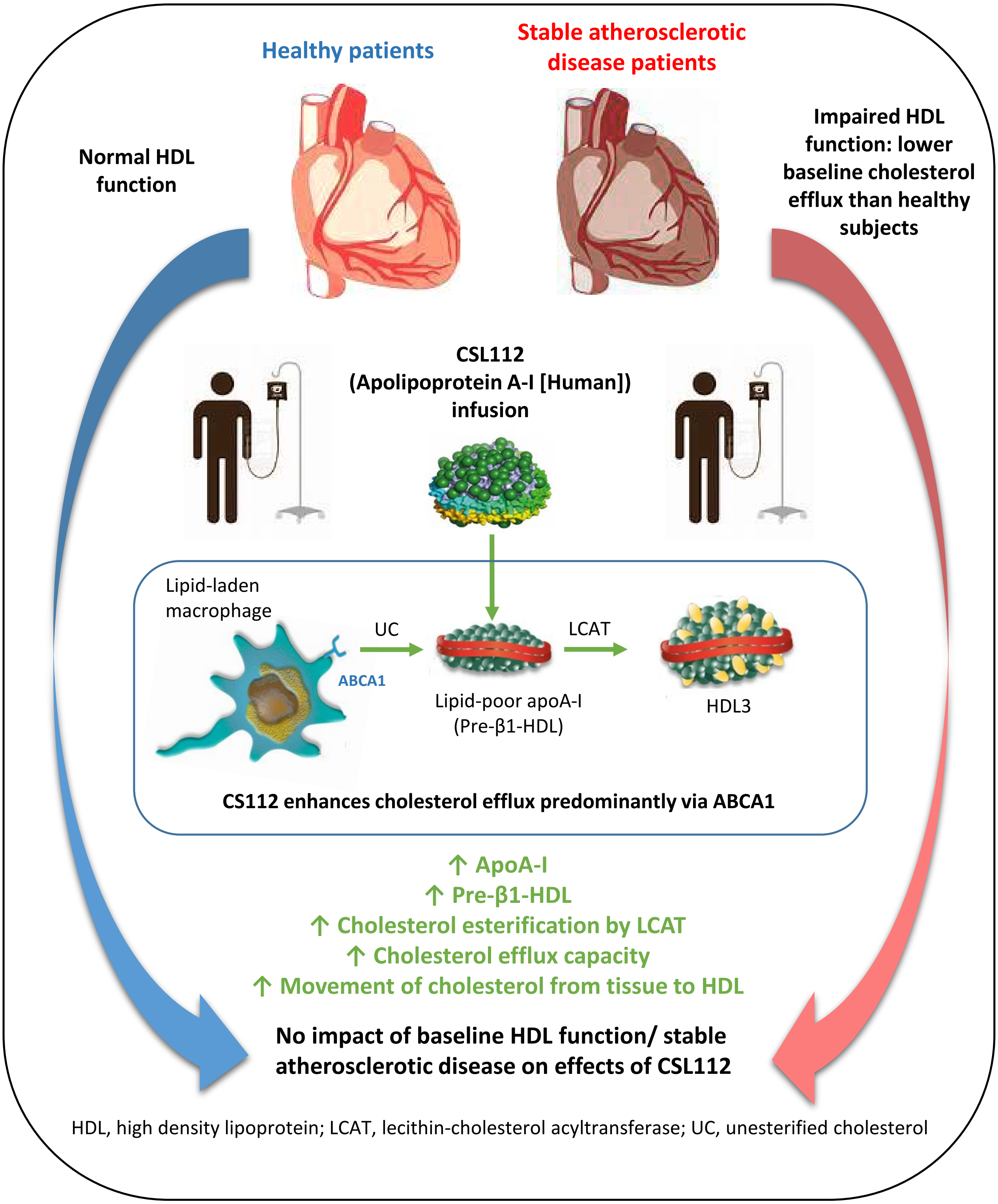
Healthy patients
Stable atherosclerotic disease patients
Normal HDL function
Impaired HDL function: lower baseline cholesterol efflux than healthy subjects
CSL112 (Apolipoprotein A-I [Human]) infusion
Lipid-laden macrophage
UC
LCAT
ABCA1
Lipid-poor apoA-I (Pre-β1-HDL)
HDL3
CS112 enhances cholesterol efflux predominantly via ABCA1
↑ ApoA-I
↑ Pre-β1-HDL
↑ Cholesterol esterification by LCAT
↑ Cholesterol efflux capacity
↑ Movement of cholesterol from tissue to HDL
No impact of baseline HDL function/ stable atherosclerotic disease on effects of CSL112
HDL, high density lipoprotein; LCAT, lecithin-cholesterol acyltransferase; UC, unesterified cholesterol
